# A qualitative study on Singaporean women’s views towards breast cancer screening and Single Nucleotide Polymorphisms (SNPs) gene testing to guide personalised screening strategies

**DOI:** 10.1186/s12885-017-3781-8

**Published:** 2017-11-21

**Authors:** Xin Yi Wong, Kok Joon Chong, Janine A. van Til, Hwee Lin Wee

**Affiliations:** 1 Department of Pharmacy, Faculty of Science, National University of Singapore, Block S4A Level 3, 18 Science Drive 4, Singapore, 117543 Republic of Singapore; 2 Department of Planning and Development, Regional Health System Planning Office, National University Health System, 1E Kent Ridge Road, Singapore, 119228 Republic of Singapore; 3 Department of Health Technology & Services Research, School for Management & Governance, University of Twente, PO Box 217, 7500 AE Enschede, The Netherlands; 4 Saw Swee Hock School of Public Health, National University of Singapore, 12 Science Drive 2, #10-01, Singapore, 117549 Republic of Singapore

**Keywords:** Breast cancer, Single nucleotide polymorphisms, Genetic testing, Personalised screening, Qualitative

## Abstract

**Background:**

Breast cancer is the top cancer by incidence and mortality in Singaporean women. Mammography is by far its best screening tool, but current recommended age and interval may not yield the most benefit. Recent studies have demonstrated the potential of single nucleotide polymorphisms (SNPs) to improve discriminatory accuracy of breast cancer risk assessment models. This study was conducted to understand Singaporean women’s views towards breast cancer screening and SNPs gene testing to guide personalised screening strategies.

**Methods:**

Focus group discussions were conducted among English-speaking women (*n* = 27) between 40 to 65 years old, both current and lapsed mammogram users. Women were divided into four groups based on age and mammogram usage. Discussions about breast cancer and screening experience, as well as perception and attitude towards SNPs gene testing were conducted by an experienced moderator. Women were also asked for factors that will influence their uptake of the test. Transcripts were analysed using thematic analysis to captured similarities and differences in views expressed.

**Results:**

Barriers to repeat mammogram attendance include laziness to make appointment and painful and uncomfortable screening process. However, the underlying reason may be low perceived susceptibility to breast cancer. Facilitators to repeat mammogram attendance include ease of making appointment and timely reminders. Women were generally receptive towards SNPs gene testing, but required information on accuracy, cost, invasiveness, and side effects before they decide whether to go for it. Other factors include waiting time for results and frequency interval. On average, women gave a rating of 7.5 (range 5 to 10) when asked how likely they will go for the test.

**Conclusion:**

Addressing concerns such as pain and discomfort during mammogram, providing timely reminders and debunking breast cancer myths can help to improve screening uptake. Women demonstrated a spectrum of responses towards a novel test like SNPs gene testing, but need more information to make an informed decision. Future public health education on predictive genetic testing should adequately address both benefits and risks. Findings from this study is used to inform a discrete choice experiment to empirically quantify women preferences and willingness-to-pay for SNPs gene testing.

**Electronic supplementary material:**

The online version of this article (10.1186/s12885-017-3781-8) contains supplementary material, which is available to authorized users.

## Background

Breast cancer is the most common cancer and one of the leading causes of deaths in women worldwide. In Singapore, it is the top cancer among women of all three main ethnic groups, Chinese, Malay and Indian. Over the years, incidence has increased almost three-fold, from 23.8 per 100,000 population between 1975 to 1979 to 64.7 per 100,000 population between 2010 to 2014 [1]. Despite rising incidence, mortality rate has remained relatively stable since the period between 1990 to 1994, due to advancement in screening, prevention as well as treatment modalities.

Mammography is considered the best screening tool for breast cancer currently and has been associated with reduction in breast cancer deaths [2]. In Singapore, average-risk and asymptomatic women between the age of 50 to 69 are recommended to go for mammogram every two years (Grade A, Level 1++) [3]. Under the national breast cancer screening programme (BreastScreen Singapore [BSS]) launched in 2002, eligible women can receive subsidised screening mammograms at government-funded polyclinics or approved private centres. Despite its relative affordability, only 11.0% of the target population aged 50 to 69 attended screening under BSS in 2008 to 2009 [4]. In a national health survey conducted in 2010, only 40.4% of women aged 50 to 59 and 38.1% of women aged 60 to 69 attended mammogram screening in the past two years [5]. To increase screening uptake, the Singapore Government has extended the use of Medisave, a compulsory medical savings scheme, to offset the cost of screening mammograms since July 2011 [4].

However, various authorities disagree with one another on the screening age and interval that will yield the most benefit for all women [6–8]. Additionally, age is just one of the many factors that influence the net benefit of screening [9, 10]. Risk calculators such as the Breast Cancer Risk Assessment Tool (BRCAT, also known as the Gail model) [11] and the Breast Cancer Screening Consortium (BCSC) Risk Calculator [12] include other risk elements (e.g. benign breast conditions, early menarche, family history etc.) are increasingly used to better stratify a woman’s risk. Some researchers have suggested these to be incorporated in screening decisions [13]. However, these decision support tools are usually limited by moderate discriminatory accuracy [14, 15].

Recent genome-wide association studies have discovered multiple common genetic variants, or Single Nucleotide Polymorphisms (SNPs) in the population, that are associated with breast cancer [16–21]. Research has shown that when combined into a polygenic risk score (PRS), these SNPs can improve discriminatory accuracy of existing risk assessment models [22, 23] and identify individuals in the population who have crossed clinically relevant risk thresholds [24]. PRS may thus inform personalised screening strategies. It may even predict breast cancer risk by pathological sub-type, as several studies have demonstrated the association between SNPs and specific breast tumour sub-types [25–27]. Hence, it is postulated that women at low risk for aggressive tumour sub-types may avoid the harms of screening by being screened less frequently, while high-risk women may take preventive measures and screen more frequently. However, this needs to be confirmed as we wait for the results of the first randomised controlled trial comparing risk-based screening against annual screening [28].

Owing to its novelty, little is known about women’s perception and attitude towards SNPs gene testing to guide risk-based breast cancer screening. It is crucial to include patients’ perspectives into health technology assessment (HTA) process, as the involvement of end-users broadens the perspective of assessments and recommendations provided to decision makers [29]. They provide a real world understanding of the disease condition and the benefits and risks of using health technologies [30]. Thus, we conducted a qualitative study to assess Singaporean women’s views towards breast cancer screening and SNPs gene testing to guide personalised screening strategies.

## Methods

### Eligibility and recruitment

We conducted focus group discussions to obtain views of screening-age women. English-speaking Singaporean women between 40 to 65 years old, both current mammogram users (have experienced it in the past two years) and lapsed mammogram users (have not experienced it in the past three years) were included. Only one respondent per household may participate, and each of the four focus groups must have at least two participants of minority race. To facilitate the discussion, each group should also include one or two women who have taken a genetic test before, or have some knowledge of it, or have considered taking it at some point in time. Participants were recruited by a recruitment partner of the market research company which we have commissioned to conduct the discussions. Recruiters would first reach out to their database network, which comprised a wide base of individuals across age bands, ethnicity and affluence, with the basic requirements of age (40 to 65 years old) and experience with mammograms (current and lapsed). They would be screened further via a mixture of mobile phones and landlines, to ensure that all potential participants fulfilled the recruitment criteria for the study. If they were unable to reach an individual on the first try, they would try to contact her a second time. 28 participants (seven in each group) were recruited but only 27 of them turned up. All participants provided written consent prior to participation in the discussions. The study was approved by the National Healthcare Group Domain Specific Review Board, Singapore (Reference number: 2016/00184). Sociodemographic characteristics such as age, ethnicity, marital status, occupation and household income were collected.

### Focus groups

The following four groups were formed: Group 1, women between 40 to 49 years old and have experienced mammogram in the past two years; Group 2, women between 40 to 49 years old and have not experienced mammogram in the past three years; Group 3, women between 50 to 65 years old and have experienced mammogram in the past two years and Group 4, women between 50 to 65 years old and have not experienced mammogram in the past three years. Discussions were conducted in August 2016, at the office of the market research company and lasted for two hours each. An experienced moderator utilised a structured interview guide (co-developed by the authors and market research company, see Additional file 1) and led consented participants through a dialogue surrounding breast cancer and individual screening experience, as well as perception and attitude towards SNPs gene testing. Using a show card, participants were introduced to SNPs gene testing and its function in classifying women into low, average or high risk of developing breast cancer in their lifetime. Participants were told that when combined with other information such as age, body mass index, family history and breast density, an individualised risk score can be derived to inform screening strategies. For example, women with low risk may be screened less frequently than currently recommended, while high-risk women may take preventive measures and screen more frequently. Spontaneous responses to SNPs gene testing were elicited. Women were also asked for the factors that will influence their uptake of the test. Discussions were recorded in audio-visual format and were transcribed verbatim. Participants received SGD80 to SGD100 compensation for their time and effort.

### Analysis

We conducted thematic analysis [31], aiming to identify a set of main themes that sufficiently captured the views expressed by the participants. Transcripts were reviewed and coded independently by two researchers. The initial codes were then grouped according to similarity in themes. Using constant comparison, we highlighted similarities and differences across the groups. The analysts compared their results and any differences were discussed and resolved.

## Results

### Participant characteristics

Characteristics of the women who agreed to participate in the study are illustrated in Table 1. About half of them were in their 40s, while the rest were in their 50s and 60s. Ethnicity was representative of the general population in Singapore, with at least one minority race participant in each focus group. Majority of the women are married, all of whom have children. About 67% of the women are currently working. The most common monthly household income is between SGD4000 to 4999, while the median monthly household income in Singapore is SGD7624 in 2015 [32]. Nonetheless, it appeared that younger participants (Group 1 and 2) have higher monthly household income. About half of the participants have attended mammogram screening in the past two years, as compared to 40.4% of women aged 50 to 59 and 38.1% of women aged 60 to 69 surveyed in 2010 [5].

**Table 1:** Characteristics of the participants

	Number (%) of participants				
	Group 1 (*n* = 7)	Group 2 (*n* = 7)	Group 3 (*n* = 7)	Group 4 (*n* = 6)	Total (*n* = 27)
Age (years)					
40–49	7 (100)	7 (100)	–	–	14 (52)
50–59	–	–	5 (71)	4 (67)	9 (33)
60–69	–	–	2 (29)	2 (33)	4 (15)
Ethnicity					
Chinese	5 (71)	5 (71)	5 (71)	5 (83)	20 (74)
Malay	1 (14)	2 (29)	1 (14)	–	4 (15)
Indian	1 (14)	–	1 (14)	1 (17)	3 (11)
Marital status					
Married	5 (71)	7 (100)	7 (100)	6 (100)	25 (93)
Single	2 (29)	–	–	–	2 (7)
Occupation status					
Employed	6 (86)	5 (71)	4 (57)	3 (50)	18 (67)
Homemaker	1 (14)	2 (29)	2 (29)	3 (50)	8 (30)
Retiree	–	–	1 (14)	–	1 (4)
Monthly household income (SGD), 2015 median monthly household income: SGD7624 [32]					
4000–4999	1 (14)	3 (43)	3 (43)	3 (50)	10 (37)
5000–6999	5 (71)	–	3 (43)	–	8 (30)
7000–9999	1 (14)	2 (29)	1 (14)	3 (50)	7 (26)
10,000 and above	–	2 (29)	–	–	2 (7)
Mammogram attendance					
Within past 12 months	4 (57)	–	4 (57)	–	8 (30)
Within past 24 months	2 (29)	–	3 (43)	–	5 (19)
Within past 36 months	1 (14)	–	–	–	1 (4)
Within past 48 months	–	–	–	–	–
More than 2 years ago	–	7 (100)	–	6 (100)	13 (48)

### Knowledge and attitude towards breast cancer and breast cancer screening

When asked about some of their top health concerns, women in all groups cited cancer, among other chronic diseases such as diabetes, stroke and dementia. One women in Group 1 mentioned that she is afraid of “getting any disease that is related to women”. Three out of seven women in Group 2 considered breast cancer to be their number one health fear. Some in Group 3 observed that “breast cancer is becoming more and more common” and even felt that the word ‘cancer’ is “akin to death sentence”. One women in Group 4 expressed that cancer is her number one health fear due to positive family history.

Majority of the women said that cancer is inherently present in everyone, whether it is activated or not (Table 2, Quote 1). When asked how to prevent cancer, women in all groups cited diet control, exercise and stress reduction. For diet control, they mentioned “avoid processed and preserved food”, “eat more fruits and vegetables”, “take less fried and oily food” and “take less red meat”. They engaged in a variety of exercise, such as “running”, “cycling”, “dancing”, “playing sports with my children” and “Tai-chi”. Some women thought that stress is a likely cause of cancer. One said that “some people said that a colleague of mine who has breast cancer is the stressful and paranoid type. Maybe that is the cause.” Another concurred, “my sister has cancer and she has a very stressful job.”

**Table 2:** Knowledge and attitude towards breast cancer and breast cancer screening and representative participant quotes

Quote number	Theme	Sub-theme	Quote
1	Cancer is in everyone		“Everybody has cancer inside our bodies. It is just a matter of when it gets triggered.”
2	Barriers to repeat mammography attendance	Laziness	“Just lazy, no sense of urgency. But if it happens to me, then maybe I will do it yearly or once every two years.”
3			“Actually, it is not difficult. It is just human nature to be lazy.”
4		Forgetfulness	“Sometimes we forget to do so. We tell ourselves that we will book next month and the next thing you know, the whole year is gone.”
5		Discomfort/ Pain	“I feel discomfort during the check. They put your breast on the plate and press against it. If the image is not well-taken, you need to repeat the process. I just don’t like it. It is painful.”
6			“The first time it was gentle and not painful, but the second time was very painful. So, I became a bit scared.”
7			“My first mammogram experience was a very painful one. After that, I find that it is not necessary to go. I did not go for one even after five years.”
8	Facilitators to repeat mammography attendance	Convenience	“When I called up the polyclinic, they referred me to specific locations that perform mammogram. I went to the one with appointment slot and it was very fast.”
9			“These days you can just book or cancel online, it is very convenient.”
10		Prior knowledge	“Because to me, it’s expected to be painful, given that something so hard is pressing onto your body. I know it will be over soon.”
11		Pain tolerance	“To me it’s bearable, because it’s not like you’re having Caesarean.”

All women attended mammogram screening at least once in their lifetime. Among the groups, those who experienced mammogram in the past two years (Group 1 and 3) had more positive encounters than those who have not experienced mammogram in the past three years (Group 2 and 4). The latter also admitted that they were lackadaisical in making the mammogram appointment. Women in both groups experienced pain or discomfort during mammogram but the former found it more bearable. Barriers to repeat mammography attendances include laziness to make appointment and dislike of the painful and uncomfortable process (Table 2, Quote 2 to 7).

Facilitators to repeat mammography attendances include ease of making appointments and higher tolerance of the painful and uncomfortable process (Table 2, Quote 8 to 11). Group 1 and Group 3 women were asked how they would encourage others to go for regular screening. They mentioned that it is “easier to treat in the early stage rather than late stage” and the result would “remove uncertainty” and “give you a peace of mind”, if it turns out to be negative. One women described avoiding mammogram as being “penny wise and pound foolish.” On the other hand, Group 2 and 4 women were asked what would motivate them to go for mammogram regularly. “Timely reminders” are useful and necessary, as they do not actively keep track and usually forget when the next screening should be. Recommendation from family physicians was also cited as one of the motivating factors.

### Perception of a new genetic test to predict breast cancer risk and guide screening strategies

In general, participants were receptive towards the idea of SNPs gene testing. However, their responses towards undergoing SNPs gene testing themselves can be broadly classified into three main categories. First group of women were willing to give the test a try (Table 3, Quote 1 to 4). The second group of women needed more information before they decide whether to embark on the test (Table 3, Quote 5 to 7). The last group of women were unwilling to give the test a try for various reasons (Table 3, Quote 8 to 10).

**Table 3:** Perception of a new genetic test to predict breast cancer risk and guide screening strategies and representative participant quotes

Quote number	Theme	Sub-theme	Quote
1	Women who were willing to give SNPs gene testing a try	Empowerment of knowledge	“Breast cancer is a common type of cancer in women, so it would be good to find out where you stand in terms of risk.”
2			“For people like me who tend to delay my appointments, this is good. If I know I am high risk, I may go more regularly.”
3			“You can decide what to do after knowing your risk profile. Gives me information and advice and I can make my decision thereafter.”
4		Practical considerations	“I think this saves time and money. If you have low risk, there is no point in screening so often.”
5	Women who needed more information before they can decide	Cost	“I need to know the cost. If it is free or cheap, I don’t mind. If it is costly, then I will not bother.”
6		Reliability	“How reliable is the test? I need to know that first.”
7			“Is the test based on European cohort? I need to know whether it is applicable to our Asian setting.”
8	Women who were unwilling to give SNPs gene testing a try	Competing priorities	“To me, it’s 50–50 because I want to focus on solving existing medical problems first before I tackle another health issue.”
9		Lack of belief	“I will just go for mammogram because you will still need to do it regardless of the results of this test.”
10		Lack of understanding of the test	“I find it very cumbersome to go through so many questions on your lifestyle and family history. Seems complicated.”

### Factors that influence women’s uptake of SNPs gene testing

When asked what were some of their concerns regarding the test, women mentioned four main factors that will influence their uptake. Younger women (40 to 49 years old, Group 1 and 2) placed highest importance on invasiveness of the test, followed by accuracy, cost and side effects. Older women (50 to 65 years old, Group 3 and 4) rank accuracy of the test as most important, followed by cost, invasiveness and side effects.

- Accuracy:
  Accuracy of the test was defined by the moderator as “how accurate the SNPs gene test is, in assessing your risk of developing breast cancer.” Women deemed accuracy in predicting risk of developing breast cancer as important, to ensure that the money spent and time invested is well-justified. Women generally have high expectations of accuracy, as they expected no less than 90% test accuracy before they would consider to take up the test.
- Cost:
  Women tend to benchmark cost of gene testing against the cost of mammogram, taking into consideration test frequency. For instance, some women would prefer to go for mammogram at the subsidised amount of SGD50 every one to two years, rather than the test if it is much more expensive and needs to be taken every few years. The maximum amount that older women were willing to pay range from SGD30 (“cost of a flu injection”) to SGD120. Younger women were willing to pay more, ranging from about SGD50 to SGD200. Some women shared that they will go for the test if it can be paid using Medisave, a national medical savings scheme where individuals put aside part of their income in the account. Since SNP gene testing will probably be performed once in a lifetime, good uptake can be expected if it is priced below SGD200.
- Invasiveness:
  The degree of invasiveness of the test was also a major concern for all women, because that will directly imply how painful the procedure may be. Some women were under the impression that blood test is inherently more accurate than a buccal swab or saliva test. However, when told that the latter will give the same accuracy in genetic test, most women prefer buccal swab. Some women were not comfortable with saliva sample collection as it makes them feel “disgusted.” A handful of women will stick with finger prick to obtain blood sample, as they find it painless, “just like how they test your blood sample for your sugar level in diabetes.”
- Side effects:
  Women in all groups were eager to know if there are any side effects, or risks associated with the test procedure. However, a couple of women remarked that “if it is just taking blood, there won’t be any side effect.”
  Other factors that women took into consideration included waiting time for results and frequency. When asked about their preference for location of pre-test discussion and the test itself, older women chose polyclinics as it renders an acceptable level of service at a lower cost. The polyclinic is a government subsidised primary care facility. General practitioners (GP) or family physicians were also deemed as acceptable options. In contrast, younger women preferred to speak with specialists at hospitals. Hospitals also have the added benefit of having facilities to do other types of tests, if these are required. With regards to discussion of test results, most women felt that a normal GP or polyclinic doctor should suffice, if the risk is low to average. However, if it is high risk, it will be better to speak to an oncologist. Preference also depends on the cost of the test. If the cost is high, women expected to be attended by an oncologist.

### Likelihood of going for SNPs gene testing

Women were asked, on a scale of 1 to 10, how likely they will go for the test, given that it is reasonably accurate, can be performed using buccal swab or finger prick and the cost is within the range that they are willing to pay. On average, women gave a rating of 7.5 (range from 5 to 10), which indicated high acceptability.

## Discussion

In this present study to evaluate the knowledge and attitude of women towards SNPs gene testing for breast cancer, our study participants demonstrated high acceptability for the novel test. Although there are numerous studies on public perception, knowledge and attitude towards personal genome testing [33–36], very few are focused on the topic of SNPs for breast cancer [37]. Our study adds to the limited literature, specifically on the spectrum of the responses towards SNPs gene testing and factors that influence its uptake, particularly from an Asian perspective.

Despite widespread awareness and fear of breast cancer, not all women in our focus groups attended mammogram screenings regularly. Although participants attributed it to pain and discomfort of the process and laziness on their part, the underlying reason seems to be their perception of individual risk or likelihood of developing breast cancer. Women with positive family history generally go for health screenings more regularly because they have been told that they may develop the same disease. On the other hand, women without family history do not perceive themselves to be at risk, or have low perceived susceptibility, based on the Health Belief Model [38]. However, about 70% of all breast cancers are not linked to family history and appear to be sporadic in nature [39]. Such information should be widely disseminated to the public to address misconceptions on perceived susceptibility to breast cancer. Our study has also found that providing timely reminders to screening-age women will encourage them to turn up for screening. They are also more likely to attend if their family doctors recommend it.

When first introduced to a novel test such as SNPs genetic test, participants displayed a range of acceptability towards it. Most could understand the purpose of performing such a test and raised questions to clarify doubts. Based on the theory of diffusion of innovations, which delineates the rate at which innovations are adopted [40], the group of women who were willing to try out SNPs gene test are termed as “early adopters”. These individuals are often opinion leaders in their social systems. Potential adopters look to them for advice and information about the innovation. A second group of women may be classified as “early majority” or “late majority” as they deliberated for some time before adopting the new test. Their unique position between the very early and very late to adopt makes them an important link in the diffusion process. Several of our respondents belonged to this group as they needed more information to dispel uncertainty, before committing to the test. The last group of women can be classified as “laggards”, who are the last in a system to adopt an innovation. They must be certain that the innovation will not fail before they will adopt. We observed a similar group of respondents who were generally uninterested in the test.

In our study, uncertainties that arise include accuracy, cost, invasiveness and side effects of the test. While the invasiveness and side effects of SNPs gene test should be similar to other genetic tests, information on its accuracy in predicting individual cancer risk and cost are severely lacking. More research is needed to help inform its analytical and clinical validity and utility. In addition to the abovementioned test characteristics, process characteristics such as pre-test discussion location, test location and waiting time for results are also important in their decision-making process. Preference for discussion and test location may be a function of women’s household income and personal earning power. Older women who preferred GP clinic and polyclinic are more likely to be homemakers or retirees than younger women, who preferred hospitals. Regardless of income level, women agreed that they are more willing to go for SNPs gene test if it can be paid using Medisave. In Singapore, where medical expenses are co-shared, out-of-pocket cost is a major consideration in medical decision making. Willingness-to-pay (WTP) for SNPs gene test may also be a function of income, as younger women cited higher WTP than older women.

An area of potential concern is that the women did not seem to think about the potential consequences of being classified as high risk, such as the impact on insurance coverage. This seems to reflect lower awareness of the full spectrum of potential risks associated with gene testing among Asians. One study comparing between American and Singaporean Parkinson’s disease patients and caregivers found that the American group displayed significantly more negative attitudes towards potential compromise in obtaining health and life insurance [41]. Hence, future research and development of SNPs gene testing should explore public health education to generate awareness on both benefits and risks, without creating unnecessary fear or barriers to participation.

While our study participants demonstrated high acceptability for SNPs gene test with a likelihood score of 7.5 out of 10, it is with the assumption that the test is highly accurate and reasonably priced. Also, participants who have agreed to join focus group discussions may be more health-conscious and receptive towards new health technologies as compared to the rest of the women population. As such, we should be cautious in extrapolating this results to the general public.

This study has some limitations. Women of Indian ethnicity were not represented in Group 2 while women of Malay ethnicity were not represented in Group 4 due to no-show. We did not conduct multiple sessions for each age and mammogram attendance stratum as we seemed to have reached data saturation. Also, as the discussions were conducted on weekdays, there may be selection bias in that women who were not able to take leave from work could not attend. In Singapore, socioeconomic status is positively associated with the use of English language at home [42]. As we did not include non-English speaking women, our findings may not sufficiently represent views of those with lower socioeconomic status. For instance, as compared to higher-educated participants, lower-educated participants were less likely to report that they want to know about their genetic risk and to believe they could do something about their breast cancer risk [43]. There is a possibility that non-English speaking women are less acceptable of SNPs gene testing.

## Conclusion

Our study has provided valuable insight on the spectrum of Singaporean women’s views towards breast cancer screening and SNPs gene testing to predict breast cancer risk and inform screening strategies. Debunking myths that breast cancer is largely hereditary, addressing concerns such as pain and discomfort during mammogram and providing external cues such as scheduled reminders and physician recommendations may lead to increase in screening uptake. More information on the test accuracy and cost will be useful in informing women’s decision to take up SNPs gene testing. Future public health education on predictive genetic testing should adequately address both benefits and risks. Findings from this study have been used to inform the design of an ongoing discrete choice experiment to empirically quantify women preferences and WTP for SNPs gene testing and to simulate uptake.

## Additional file

Additional file 1: Identifying attributes for SNP gene testing and its appeal in identifying how often & when to go for mammograms. (PDF 500 kb)

## Abbreviations

BCSC: Breast cancer screening consortium
BRCAT: Breast cancer risk assessment tool
GP: General practitioner
HTA: Health technology assessment
SNPs: Single Nucleotide Polymorphisms

## References

[1] Singapore Cancer Registry Interim Annual Registry Report: Trends in Cancer Incidence in Singapore 2010 to 2014. National Registry of Disease Office. 2015. https://www.nrdo.gov.sg/docs/librariesprovider3/default-document-library/cancer-trends-2010-2014_interim-annual-report_final-(public).pdf?sfvrsn=0. Accessed 22 Oct 2016.

[2] Weedon-Fekjær H, Romundstad PR, Vatten LJ (2014). Modern mammography screening and breast cancer mortality: population study. BMJ.

[3] Ministry of Health, Singapore. Cancer Screening: MOH Clinical Practice Guidelines 1/2010; 2010. https://www.moh.gov.sg/content/dam/moh_web/HPP/Doctors/cpg_medical/current/2010/cpg_Cancer%20Screening%20Booklet%20FINAL%20v6.pdf. Accessed 1 Nov 2016.

[4] Loy EY, Molinar D, Chow KY, Fock C (2015). National Breast Cancer Screening Programme, Singapore. Evaluation of participation and performance indicators. J Med Screen.

[5] Ministry of Health, Singapore. National Health Survey 2010. Singapore: Epidemiology & Disease Control Division, Ministry of Health; 2010.

[6] American College of Obstetricians & Gynecologists (2011). Breast cancer screening. Practice bulletin no. 122. Obstet Gynecol.

[7] Bevers TB, Helvie M, Bonaccio E, et al. National Comprehensive Cancer Network Clinical Practice Guidelines in Oncology. Breast Cancer Screen Diagn. 2016:24–34.

[8] Siu AL (2016). Screening for breast cancer: U.S. preventive services task force recommendation statement. Ann Intern Med.

[9] van Ravesteyn NT, Miglioretti DL, Stout NK (2012). Tipping the balance of benefits and harms to favor screening mammography starting at age 40 years: a comparative modeling study of risk. Ann Intern Med.

[10] Kerlikowske K, Zhu W, Hubbard RA (2013). Et al; breast cancer surveillance consortium. Outcomes of screening mammography by frequency, breast density, and postmenopausal hormone therapy. JAMA Intern Med.

[11] Breast Cancer Risk Assessment Tool. https://www.cancer.gov/bcrisktool/. Accessed 27 Oct 2016.

[12] Breast Cancer Surveillance Consortium Risk Calculator. https://tools.bcsc-scc.org/BC5yearRisk/calculator.htm. Accessed 27 Oct 2016.

[13] Pace LE, Keating NL (2014). A systematic assessment of benefits and risks to guide breast cancer screening decisions. JAMA.

[14] Amir E, Freedman OC, Seruga B, Evans DG (2010). Assessing women at high risk of breast cancer: a review of risk assessment models. J Natl Cancer Inst.

[15] Tice JA, Cummings SR, Smith-Bindman R, Ichikawa L, Barlow WE, Kerlikowske K (2008). Using clinical factors and mammographic breast density to estimate breast cancer risk: development and validation of a new predictive model. Ann Intern Med.

[16] Cai Q, Zhang B, Sung H (2014). Genome-wide association analysis in east Asians identifies breast cancer susceptibility loci at 1q32.1, 5q14.3 and 15q26.1. Nat Genet.

[17] Fejerman L, Ahmadiyeh N, Hu D (2014). Genome-wide association study of breast cancer in Latinas identifies novel protective variants on 6q25. Nat Commun.

[18] Michailidou K, Beesley J, Lindstrom S (2015). Genome-wide association analysis of more than 120,000 individuals identifies 15 new susceptibility loci for breast cancer. Nat Genet.

[19] Zheng W, Zhang B, Cai Q (2013). Common genetic determinants of breast-cancer risk in east Asian women: a collaborative study of 23 637 breast cancer cases and 25 579 controls. Hum Mol Genet.

[20] Fletcher O, Johnson N, Orr N (2011). Novel breast cancer susceptibility locus at 9q31.2: results of a genome-wide association study. J Natl Cancer Inst.

[21] Low S-K, Takahashi A, Ashikawa K (2013). Genome-wide association study of breast cancer in the Japanese population. PLoS One.

[22] Darabi H, Czene K, Zhao W, Liu J, Hall P, Humphreys K (2012). Breast cancer risk prediction and individualised screening based on common genetic variation and breast density measurement. Breast Cancer Res.

[23] Dite GS, Macinnis RJ, Bickerstaffe A (2016). Breast cancer risk prediction using clinical models and 77 independent risk-associated SNPs for women aged under 50 years: Australian breast cancer family registry. Cancer Epidemiol Biomark Prev.

[24] Mavaddat N, Pharoah PDP, Michailidou K (2015). Prediction of breast cancer risk based on profiling with common genetic variants. J Natl Cancer Inst.

[25] Broeks A, Schmidt MK, Sherman ME (2011). Low-penetrance breast cancer susceptibility loci are associated with specific breast tumor subtypes: findings from the breast cancer association consortium. Hum Mol Genet.

[26] Stevens KN, Vachon CM, Lee AM (2011). Common breast cancer susceptibility loci are associated with triple negative breast cancer. Cancer Res.

[27] Garcia-Closas M, Chanock S (2008). Genetic susceptibility loci for breast cancer by estrogen receptor (ER) status. Clinical Cancer Res.

[28] Wisdom: About the Study. https://wisdom.secure.force.com/portal/WsdSiteStudy. Accessed 22 Oct 2016.

[29] Hailey D, Nordwall M (2006). Survey on the involvement of consumers in health technology assessment programs. Int J Technol Assess Health Care.

[30] Facey K, Boivin A, Gracia J (2010). Patients’ perspectives in health technology assessment: a route to robust evidence and fair deliberation. Int J Technol Assess Health Care.

[31] Thomas J, Harden A (2008). Methods for the thematic synthesis of qualitative research in systematic reviews. BMC Med Res Methodol.

[32] Household Income – Tables. http://www.singstat.gov.sg/statistics/browse-by-theme/household-income-tables. (Table 18A. Average and Median Monthly Household Income from Work (Excluding Employer CPF Contributions) Among Resident and Resident Employed Households, 2000–2015). Accessed 27 Oct 2016.

[33] Leighton JW, Valverde K, Bernhardt BA (2011). The general public’s understanding and perception of direct-to-consumer genetic test results. Public Health Genomics..

[34] Savard J, Mooney-Somers J, Newson AJ, Kerridge I (2014). Australians’ knowledge and perceptions of direct-to-consumer personal genome testing. Intern Med J.

[35] Vayena E, Gourna E, Streuli J, Hafen E, Prainsack B (2012). Experiences of early users of direct-to-consumer genomics in Switzerland: an exploratory study. Public Health Genomics..

[36] Gray SW, Hicks-Courant K, Lathan CS, Garraway L, Park ER, Weeks JC (2012). Attitudes of patients with cancer about personalized medicine and somatic genetic testing. J Oncol Pract.

[37] Graves KD, Peshkin BN, Luta G, Tuong W, Schwartz MD (2011). Interest in genetic testing for modest changes in breast cancer risk: implications for SNP testing. Public Health Genomics..

[38] Champion VL, Skinner CS, Glanz K, Barbara KR, Viswanath K (2008). The health belief model. Health behaviour and health education: theory, research and practice.

[39] Ellsworth RE, Decewicz DJ, Shriver CD, Ellsworth DL (2010). Breast cancer in the personal genomics era. Curr Genomics.

[40] Rogers EM (2003). Innovativeness and adopter categories. Diffusion of innovation.

[41] Tan EK, Lee J, Hunter C, Shinawi L, Fook-Chong S, Jankovic J (2007). Comparing knowledge and attitudes towards genetic testing in Parkinson’s disease in an American and Asian population. J Neurol Sci.

[42] Statistics Singapore. Census of Population 2000, Advance Data Release. In Chapter 4: Literacy and language. https://www.singstat.gov.sg/docs/default-source/default-document-library/publications/publications_and_papers/cop2000/census_2000_advance_data_release/chap4.pdf. Accessed 15 July 2017.

[43] Bowen DJ, Harris J, Jorgensen CM, Myers MF, Kuniyuki A (2010). Socioeconomic influences on the effects of a genetic testing direct-to-consumer marketing campaign. Public Health Genomics.

