# Supplementary material for: A qualitative study on Singaporean women’s views towards breast cancer screening and Single Nucleotide Polymorphisms (SNPs) gene testing to guide personalised screening strategies
Source: BMC Cancer. 2017 Nov 21;17:776. doi: 10.1186/s12885-017-3781-8 (PMC5697412; doi:10.1186/s12885-017-3781-8)
Supplement: Additional file 1: — Identifying attributes for SNP gene testing and its appeal in identifying how often & when to go for mammograms. (PDF 500 kb) [file 12885_2017_3781_MOESM1_ESM.pdf]

PROJECT ICE SCREEN: IDENTIFYING ATTRIBUTES FOR SNP GENE TESTING AND ITS APPEAL IN IDENTIFYING HOW OFTEN & WHEN TO GO FOR MAMMOGRAMS

DISCUSSION GUIDE FOR FOCUS GROUPS
VERSION 2: 28TH JULY 2016

1. Introduction and Warm Up [10 MINS]

- Introduce self & market research company
- Purpose of Market Research and this session – to obtain personal views & opinions, so feel free to speak up.
- Informal session lasting 2 hours
- No right or wrong answers
- Views and identity to be kept confidential
- Audio-recorder as substitute for note taking.
- Introduce yourself: tell us your name, hobbies, and something that not many people know about you.

2. Setting the context [20 MIN]

Today, we are interested to talk about health…

- When you think about health, what comes to mind?
- What is considered 'good health' to you? [moderator to list on the flip chart]
    - Do you go for health checks or medical screenings?
    - Which ones do you go for? When and how often do you for these?
- And now, what are the fears that you have about health?
    - [If not mentioned] What about cancers?
    - [If not mentioned] What about breast cancer, is it one of your concerns?
- For each fear/concern mentioned earlier:
    - Why are you concerned about this?
    - When did you start having this concern? How did it get started?
    - What are you specifically concerned about?
    - Has this concern changed over time – say from 5 years ago vs. today?
    - What have you done about these health concerns that you have?
        - Are there any prevention methods that you have undertaken? Which ones, and why?

I'd like to understand a little bit more about breast cancer, please share with me what you know about it.

- What do you know about breast cancer?
    - Are there certain people who are more susceptible to it? Who?
    - Are there symptoms of breast cancer? What are some of the signs of breast cancer?
    - What are the ways to check or screen for breast cancer?
        - For each way: Do you do this? How often?
    - Do you know of ways to prevent breast cancer? How?

        - For each way: Do you do so? How often?
    - Are there cures for breast cancer? How do you do that?
- When it comes to breast cancer, is there anything you'd like to know more about?

3. Perceptions of genetic testing in general & reactions to the explanation of general genetic testing [30 MIN]

Now I'd like to understand your perceptions on genetic testing in general.

- Has anyone heard about genetic testing in general? What have you heard?
- When I say genetic testing, what comes to your mind? Why?
- What do you associate with genetic testing? **Moderator to make respondents list as many examples as possible**
- When you think about genetic testing, how does it make you feel? What emotions do you associate with it?
- What are the advantages or benefits of going for a genetic testing?
- How about the disadvantages or negative aspects of genetic testing?
- Have you ever thought about going for genetic testing? Can you explain why? For those who have heard about genetic testing and have not gone for it yet, what are the reasons?

Moderator to now show the general gene testing stimulus to the respondents and check:

Spontaneous reaction overall

- First impressions? What do you think about it?

Appeal

- What do you like about it?
- What do you dislike about it?

Needs fulfilment

- Does this feel relevant to you?
- Do you think genetic testing fulfils a need? Why?
- What concerns do you have with the kinds of tests mentioned? Why?

4. Understanding linkage of gene testing to breast cancer and ideal breast cancer screening programme [15 MIN]

- Do any one of you know that gene testing can help provide information about when and how often to go for mamograms? If yes, what do you know about it?
- Talking about breast cancers - If you could create the ideal breast screening test, what would it be like?
- Why is this ideal?
    - How is this different from the current mammogram process in the market?
    - Would it get you to go for breast screening more regularly? Why?

5. Evaluation of the SNP Gene testing idea [30 MIN]

We know mammograms are the most common breast cancer screening method. But there is a step before Mamograms which can tell us when and how often to go for mammograms depending on our breast cancer risk. It is called SNP gene testing.

- Have you heard of it before?
- What have you heard about it?
- Where did you hear about it from?

Here is a quick description of what SNP gene testing is about. Please note that this is not an ad, but to share the background information of SNP gene testing and its potential to estimate breast cancer risk, for you to have a better understanding of when and how often to go for mamograms

[Moderator to show the SNP Gene testing idea and ask the following]

Spontaneous reaction overall

- First impressions? What do you think about it?

Appeal

- What do you like about it?
- What do you dislike about it?

Needs fulfilment

- Does this feel relevant to you?
- Do you think SNP gene testing fulfils a need? Why?
- What concerns do you have with this testing? Why?
- Do you know that genetic tests may also be used for other uses, for example to determine the risk of developing an adverse drug reaction or to determine the likelihood that one will respond to a treatment?
    - Is this interesting or appealing to you?
    - Would you use it?
    - Would your concerns over the genetic tests vary for these other uses of genetic tests?

Call to action

- Given the background information on SNPs and its potential to estimate breast cancer risk, would you go for SNPs gene testing if it were available? Would you want to try it out to know when and how often you should go for mamogramms
    - Which aspects of the idea make you feel like trying out SNP gene testing? Why?

6. Evaluation of the SNP gene testing process [30 MIN]

Now that you have an understanding of what SNP gene testing is about, I'd like to understand what you think of the various aspects of the testing process.

- **UNAIDED PROBING FOR FACTORS** - Based on what I've shown you, what are the different factors that would influence you in going for the SNP gene testing? For each factor:
    - How would this affect your decision to go for the test? Why? **MODERATOR TO GET AS MANY FACTORS AS POSSIBLE AT AN UNAIDED LEVEL**
- **Pre-test discussion**
    - Is it important to have a person who discusses the pros and cons of testing before you go for the test? Why?
    - Who would you like the discussion to be with? (e.g. genetic counselling nurse, GP, specialist, etc.)
- **Waiting time**
    - What would be a reasonable amount of time that you would be willing to wait for results?
- **Accuracy of the test**
    - Are you aware of how accurate other breast screening programmes or mammograms are in assessing breast cancer risk?
    - How accurate would you expect this test to be?
- **DNA collection method**
    - This test requires a collection of DNA sample from you. Which would be acceptable to you? Which would you prefer most?
        - Dried blood spot: a spot of blood obtained from a fingerprick and blotted onto a filter paper
        - Saliva: a sample of saliva collected in a test tube
        - Buccal swab: a swab of the inside of the cheek using cotton bud)
- **Location of the testing**
    - Is location of the test important to you? Why?
    - Where would you expect the SNP gene testing to be done? (e.g. GP clinic, polyclinic, hospital, mobile clinics in the form of vans, etc.)
- **Cost of SNP gene testing**
    - How much do you think a one-time SNP gene testing would cost you? What would be a reasonable amount or range to pay for it?
    - What are the different factors that would impact cost? (e.g. which hospital/clinic it is administered in, depth of diagnosis, etc.)
- Which of the above factors are an important influence in getting you to go for the testing? Why?
- What else can be done, or what other characteristics should the test have, to encourage you to go for SNP gene testing?

- If it were part of a health screening bundle/package, would it get you to go for it? Why?

Call to action

- Given the more specific details on SNP gene testing, would you go for SNPs gene testing if it were available? Why?
    - Which aspects of the idea make you feel like trying out SNP gene testing? Why?
- Do you read up on health, health issues, etc.? Do you actively seek information?
    - Where do you go to find out information? Or where do you remember coming across information?
    - If we wanted to tell you more about SNP gene testing, which channel would be most effective in reaching out to you?

7.Experience with mammograms [20 MIN]

Now, I'd like to hear about your experiences with mammograms, and how you decide whether to go for it.

Current mammogram users:

- How often do you usually go for a mammogram?
- Can you recall the first time you went for a mammogram? What was the reason? Could you describe the experience to me?
- Why do you go for mammograms regularly? Apart from your own motivations, what are the factors in the screening programme you use that keep you motivated?
- What are the factors that motivate or encourage you to go for mammograms on a regular basis?
- If we wanted to encourage those who don't go for mammograms to do so, what advice or information would you share with them?
- What do you think is good about going for mammograms, that perhaps people who don't regularly go for mammograms may not know about?
- What do you dislike about going for mammograms? What is one thing that you would like to change about the mammogram process?

Lapsed mammogram users:

- How often did you go for mammograms?
- Can you describe your first mammogram experience? What did you like and dislike about it?
- Why do you not go for mammograms more regularly?
- Apart from your own internal motivations, what were those critical factors in the mammogram experience which dissuaded you to continue them further?
- Do you other forms of breast screening instead?
    - Why do you do those instead?
    - How are they better than mammograms?
- What are the factors that discourage you from going for mammograms on a more regular basis?
- What could be done to get you to go for mammograms more regularly?

- [If pricing is mentioned] What else, aside from price could we do to encourage you to go for a mammogram instead?

Among all:

- When was your last mammogram?
  - Why did you go for that mammogram? What triggered you to go? (e.g. part of a health package, someone I knew had breast cancer, etc.)
  - How much did you pay for it?
  - Was your mammogram part of a package, and if so what else was included in the package? How much was the package?
- What was the experience like? Please share both the positive and negative aspects of the mammogram experience across the various touchpoints.
  - Making an appointment (how the appointment was made, waiting time, location, etc.)
  - Interaction and advice from the doctor (bedside manner, advice given, time spent with you, etc.)
  - The mammogram experience (the process, level of discomfort, attention received from doctor/nurse, etc.)
  - Post-mammogram experience (waiting time for test results, accuracy, etc.)
    - Have you experienced a false positive for any of your tests? What was that experience like?
    - How long did you have to wait for your breast screening results?

7. Wrap-up [5 MIN]

Exploring any other thoughts and closing the session

- Ask any additional questions
- [Moderator to check if there are any extra questions from client]
- Close the session and thank respondents
